# A Soft, Flexible Implant for Wireless Photothermal–Pyroelectric Neurostimulation

**DOI:** 10.1002/advs.202519616

**Published:** 2026-01-20

**Authors:** Jiang Wu, Minmin Mao, Beltzane Garcia Cirera, Hao Ye, Xiangzhong Chen, Josep Puigmartí‐Luis, Ni Qin, Salvador Pané

**Affiliations:** ^1^ School of Electronic Science and Engineering (School of Microelectronics) South China Normal University Foshan China; ^2^ College of Electronic Information and Engineering Hangzhou Dianzi University Hangzhou China; ^3^ Institute of Robotics and Intelligent Systems ETH Zurich Zurich Switzerland; ^4^ Departament De Ciència dels Materials i Química Física and Institut de Química Teòrica i Computacional University of Barcelona Barcelona Spain; ^5^ ICREA Institució Catalana de Reserca i Estudis Avançats Barcelona Spain; ^6^ State Key Laboratory of Optoelectronic Materials and Technologies School of Materials Science and Engineering Sun Yat‐Sen University Guangzhou China; ^7^ International Institute For Intelligent Nanorobots and Nanosystems College of Intelligent Robotics and Advanced Manufacturing State Key Laboratory of Photovoltaics and Perception Shanghai Frontiers Science Research Base of Intelligent Optoelectronics and Perception Institute of Optoelectronics Fudan University Shanghai China; ^8^ Zhejiang Key Laboratory of Extreme Environment Functional Materials Yiwu Research Insititute of Fudan university Yiwu China

**Keywords:** BaTiO_3_, cell differentiation, photothermal, pyroelectric

## Abstract

Neural progenitor cells (NPCs) are widely recognized as promising seed cells for the treatment of neurodegenerative diseases due to their inherent potential to differentiate into functional neurons. However, due to the low differentiation efficiency, their practical application in neural repair remains a significant challenge. In this work, we present a novel, non‐invasive strategy to enhance the neuronal differentiation of NPCs through photothermal‐pyroelectric stimulation. Specifically, a hybrid film was fabricated by coating BaTiO_3_ (BTO) nanocrystals – comprising either nanoparticles or nanosheets – onto the surface of a CNT@PDMS composite membrane. Under periodic light irradiation, the photothermal effect triggered rapid temperature oscillations, which activated BTO's pyroelectric effect, generating localized electric stimulation that promoted neuronal differentiation of NPCs. Immunofluorescent staining and Western blot analysis further confirmed that the observed enhancement in neural differentiation was mediated by the activation of Ca^2+^ signaling and the PI3K/Akt pathway. Moreover, comparative experiments revealed that BTO nanosheets with highly exposed (001) facets exhibited markedly enhanced neurogenic differentiation capabilities relative to BTO nanoparticles, highlighting the facet‐dependent pyroelectric effect. This work presents wireless photothermal‐pyroelectric stimulation for neural differentiation and, for the first time, proposes a pyroelectric regenerative medicine for the non‐invasive repair of damaged neural tissue.

## Graphical abstract

A replication strategy is used to enlarge the exposed surface area of barium titanate within flexible films with embedded carbon nanotubes, enabling efficient photothermal‐pyroelectric stimulation. Polar crystalline nanosheets show superior performance in guiding neural progenitor cells toward neuronal differentiation. These findings introduce pyroelectric regenerative medicine as a promising and minimally invasive approach to neural tissue repair.

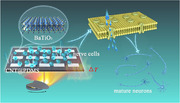

## 1 Introduction

Traumatic injuries and neurodegenerative diseases of the central nervous system can cause irreversible damage to neuronal networks and severe motor impairment [1, 2]. Although researchers and clinicians have made significant efforts and achieved substantial progress [3], achieving functional neural regeneration remains a major clinical challenge because mature neurons have limited proliferative capacity [4]. Neural tissue engineering (NTE) has emerged as a promising strategy that integrates biomaterial scaffolds, biochemical/biophysical cues, and neural stem or progenitor cells to promote neuronal differentiation, rebuild circuits, and restore function [5]. The NTE process primarily involves three key components: seeded cells, scaffolds, and growth factors, with scaffolds playing a crucial role in regulating cell differentiation and promoting tissue functionalization [6]. Among various stem cell types, neural progenitor cells (NPCs) are considered promising candidates for NTE because of their ability to release neurotrophic factors and modify the immune environment [7]. Building on this, researchers have proposed various strategies involving the development of growth factors to enhance the engraftment and differentiation of transplanted NPCs [8]. However, due to short half‐lives and potential side effects in physiological environments [9], growth factor‐based strategies still struggle to meet practical application requirements.

In recent years, electrical stimulation has emerged as a promising therapeutic strategy for enhancing neural differentiation of NPCs [10, 11]. Accumulating evidence demonstrates that electrical stimulation promotes multiple neurogenic processes in vitro, including stem cell migration, neuronal differentiation, neurite outgrowth, and modulation of intracellular Ca^2+^ dynamics [12]. However, conventional electrical stimulation methods, such as direct current (DC) and alternating current (AC) approaches, face significant limitations due to their reliance on wired systems and the necessity for external power sources [13, 14, 15]. Therefore, wireless stimulation technologies were proposed to overcome the limitations associated with invasive wired systems. In this vein, energy‐converting materials, especially ferroelectric nanomaterials, have attracted considerable research interest owing to their unique capability of generating electrical signals wirelessly in response to external stimuli [16, 17]. A prominent example is BaTiO_3_ (BTO) [18], which exhibits dual stimulation modalities: ultrasonic vibrations induce a piezoelectric response, whereas temperature fluctuations trigger a pyroelectric response. While ultrasound‐mediated piezoelectric stimulation has been experimentally demonstrated to enhance both the rate and efficiency of neural cell differentiation into mature neurons [19, 20], the potential risk of additional damage to injured tissues caused by high‐intensity ultrasound remains a challenge in certain scenarios [21]. In contrast, pyroelectric stimulation, driven by non‐contact thermal fluctuations, offers enhanced biocompatibility and represents a promising alternative. Given that the pyroelectric effect is highly dependent on temperature fluctuations, activation of pyroelectricity by means of the photothermal effect is a promising approach to generating rapid temperature variations [22], thereby enhancing the pyroelectric response.

Here, we present a soft, flexible scaffold that delivers wireless electrical stimulation via photothermal–pyroelectric coupling in a CNT@PDMS/BTO trilayer, where PDMS stands for polydimethylsiloxane, and CNT for carbon nanotubes. Unlike prior pyroelectric flexible films [23], in which BTO is deeply embedded within the membrane, restricting its direct interaction with the external environment, a simple “replication process” enables the arrangement of BTO nanocrystals, including either nanoparticles or nanosheets, on the surface of a flexible PDMS film. This configuration results in the formation of a PDMS/BTO bio‐scaffold with enhanced surface accessibility. Surface localization retains BTO nanocrystals on the scaffold and increases its effective exposure, improving stimulation efficiency. Under periodic light illumination, the CNT layer converts light to heat, producing temperature transients that drive pyroelectric output at the scaffold surface. In the absence of any biological or chemical differentiation factors, gene‐ and protein‐expression analyses confirm that the pyroelectric stimulates alone is sufficient to induce neural differentiation of NPCs. In contrast, NPCs cultured on CNT@PDMS films subjected solely to thermal stimulation, or on CNT@PDMS/BTO films without thermal inputs, show no or minimal differentiation. These findings establish a novel, wireless, and biocompatible approach for promoting neuronal differentiation, highlighting its potential applications in autologous stem cell therapy for neurodegenerative diseases.

## 2 Results and Discussion

**FIGURE 1:**
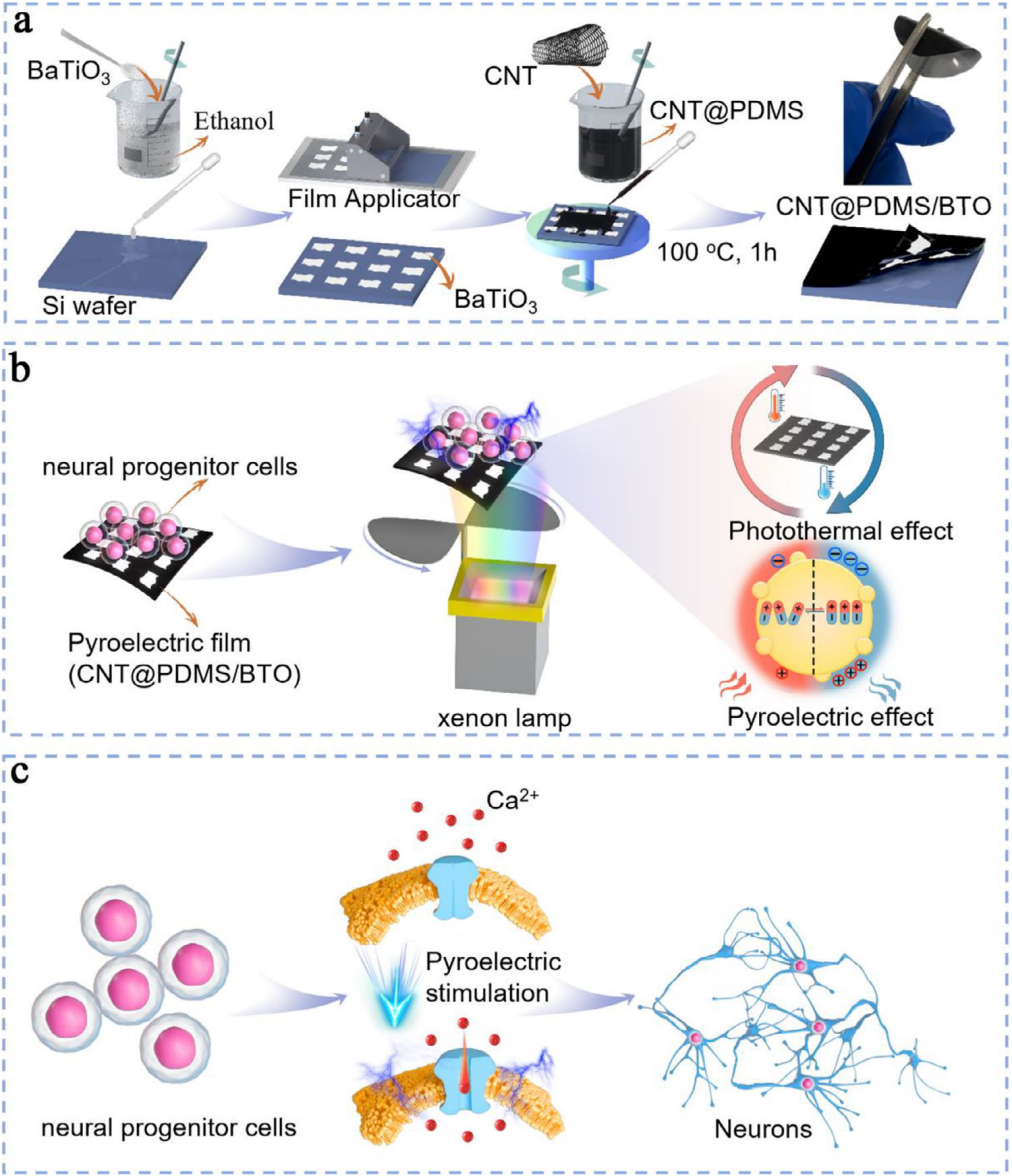
The schematic representation of the work: (a) The preparation process of CNT@PDMS/BTO scaffold; (b) Evaluation of photothermal‐pyroelectric effect on neural promoter cells induced by the periodic illumination on CNT@PDMS/BTO scaffold; (c) The possible mechanism of pyroelectric stimulation on the differentiation of neural promoter cells.

Spin coating methodology was employed to prepare the inorganic–organic composite CNT@PDMS/BTO scaffolds due to its ability to easily control the thickness of the PDMS layer. As illustrated in Figure 1a, hydrothermally synthesized BTO nanosheets/nanoparticles were uniformly dispersed in ethanol and deposited onto silicon wafers via doctor blade coating. Following ethanol evaporation, a PDMS solution containing CNTs was spin‐coated onto the BTO‐modified silicon substrate. After thermal curing, the CNT@PDMS composite film with surface‐embedded BTO was carefully peeled from the silicon wafer, resulting in the desired CNT@PDMS/BTO scaffolds. The proposed photothermal‐pyroelectric neural differentiation platform is schematically illustrated in Figure 1b, where NPCs were cultured on the BTO‐exposed surface while periodic light illumination was applied to the opposite side. In this configuration, the CNTs harness photothermal conversion to generate rapid thermal fluctuations that propagate through the BTO component, establishing localized pyroelectric stimulation. Similar to piezoelectric stimulation [24], the pyroelectric stimulation of BTO is expected to promote the differentiation of surface‐adhered NPCs into mature neurons, as illustrated in Figure 1c.

**FIGURE 2:**
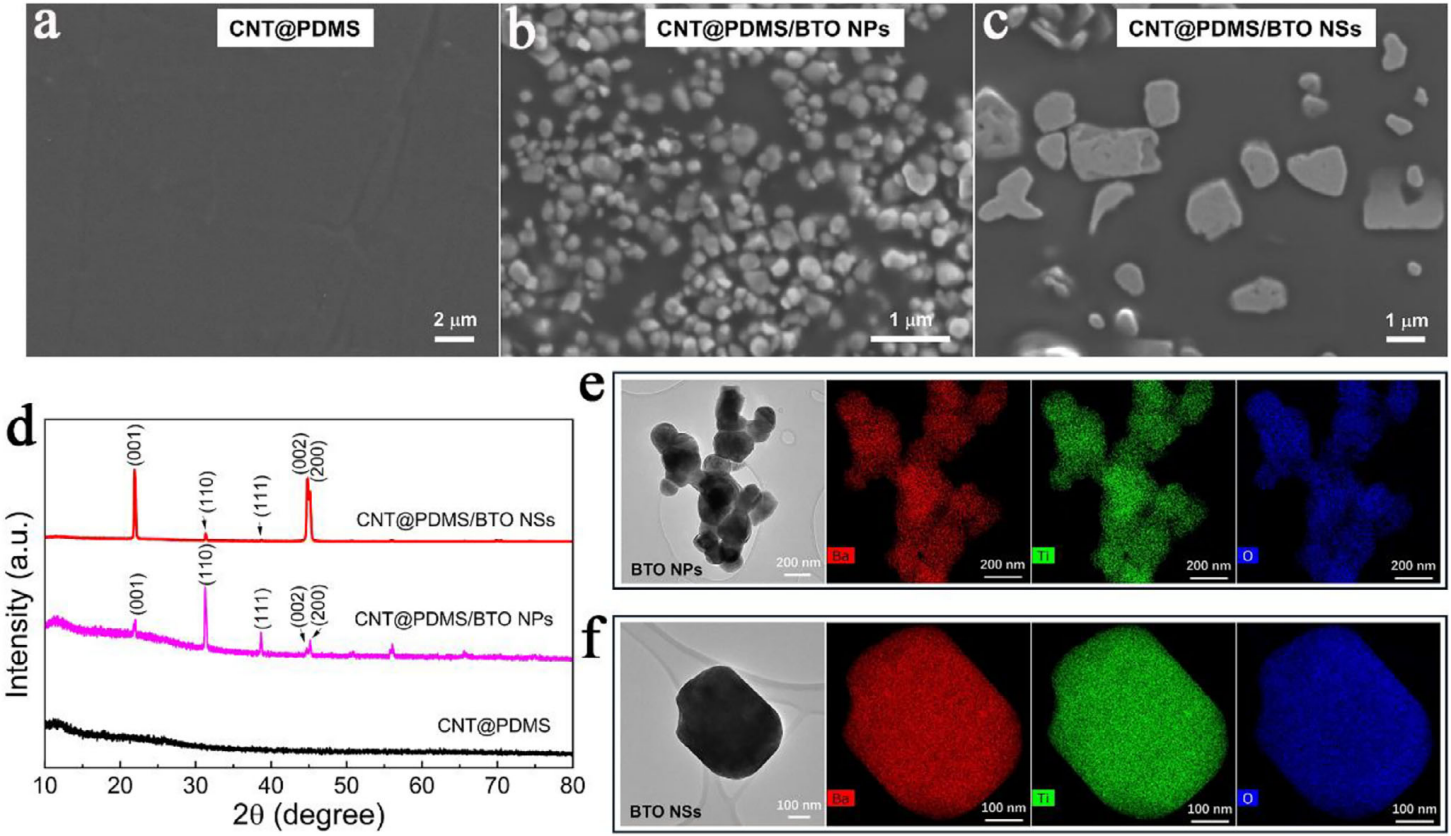
The SEM images of (a) CNT@PDMS, (b) CNT@PDMS/BTO NPs, and (c) CNT@PDMS/BTO NSs. (d) XRD patterns of CNT@PDMS, CNT@PDMS/BTO NPs, and CNT@PDMS/BTO NSs. TEM images and EDS mapping of (e) BTO nanoparticles and (f) BTO nanosheets.

The surface morphologies of (i) bare CNT@PDMS, (ii) CNT@PDMS incorporating BTO nanoparticles loaded (abbreviated as CNT@PDMS/BTO NPs), and (iii) CNT@PDMS incorporating BTO nanosheets (CNT@PDMS/BTO NSs) were examined by scanning electron microscopy (SEM), as depicted in Figure 2a–c. The bare PDMS surface exhibits smooth morphology without discernible wrinkles, whereas the surfaces of CNT@PDMS/BTO composites appear uneven due to the incorporation of BTO. Benefiting from the pre‐dispersion of BTO samples onto the Si substrate, the BTO nanoparticles were uniformly distributed and firmly anchored on the surfaces, instead of being embedded within the PDMS matrix [23]. The BTO nanoparticles display a relatively dense and randomly distributed arrangement, with particle sizes ranging from tens to hundreds of nanometers. In contrast, the BTO nanosheets predominantly adopt a “lying flat” orientation on the surface of CNT@PDMS film surfaces, with exposed surface areas measuring several hundred nanometers in dimension. The surface morphological features at a larger scale can be found in Figure S1.

To investigate the crystalline structure of the synthesized CNT@PDMS/BTO composite scaffold, X‐ray diffraction (XRD) analysis was conducted. The CNT@PDMS control sample exhibited no discernible diffraction peaks, consistent with the amorphous nature of the PDMS matrix and the disordered distribution of CNTs within the polymer. Upon BTO loading, well‐defined diffraction peaks corresponding to the pure tetragonal phase (JCPDS 05–0626) were clearly resolved. A pronounced difference in crystallographic orientation was observed between BTO nanoparticle and nanosheet composites. The CNT@PDMS/BTO NPs sample exhibited a strongly predominant (110) diffraction peak, while in the CNT@PDMS/BTO NSs sample, the (110) reflection was substantially diminished, with the (001) and (002) reflections emerging as dominant peaks. This distinct diffraction profile, coupled with SEM observations of horizontally aligned BTO nanosheets, strongly suggests that the BTO nanosheets preferentially expose (001) crystallographic planes, with their thickness direction oriented along the [001] axis [24]. The morphological characteristics of BTO samples were further investigated using transmission electron microscopy (TEM). As shown in Figure 2e,f, the TEM micrographs clearly reveal well‐defined particulate and plate‐like nanostructures. Corresponding energy‐dispersive X‐ray spectroscopy (EDS) elemental mapping demonstrated a homogeneous spatial distribution of Ba, Ti, and O elements throughout the sample matrix, confirming the compositional uniformity of the synthesized BTO samples.

**FIGURE 3:**
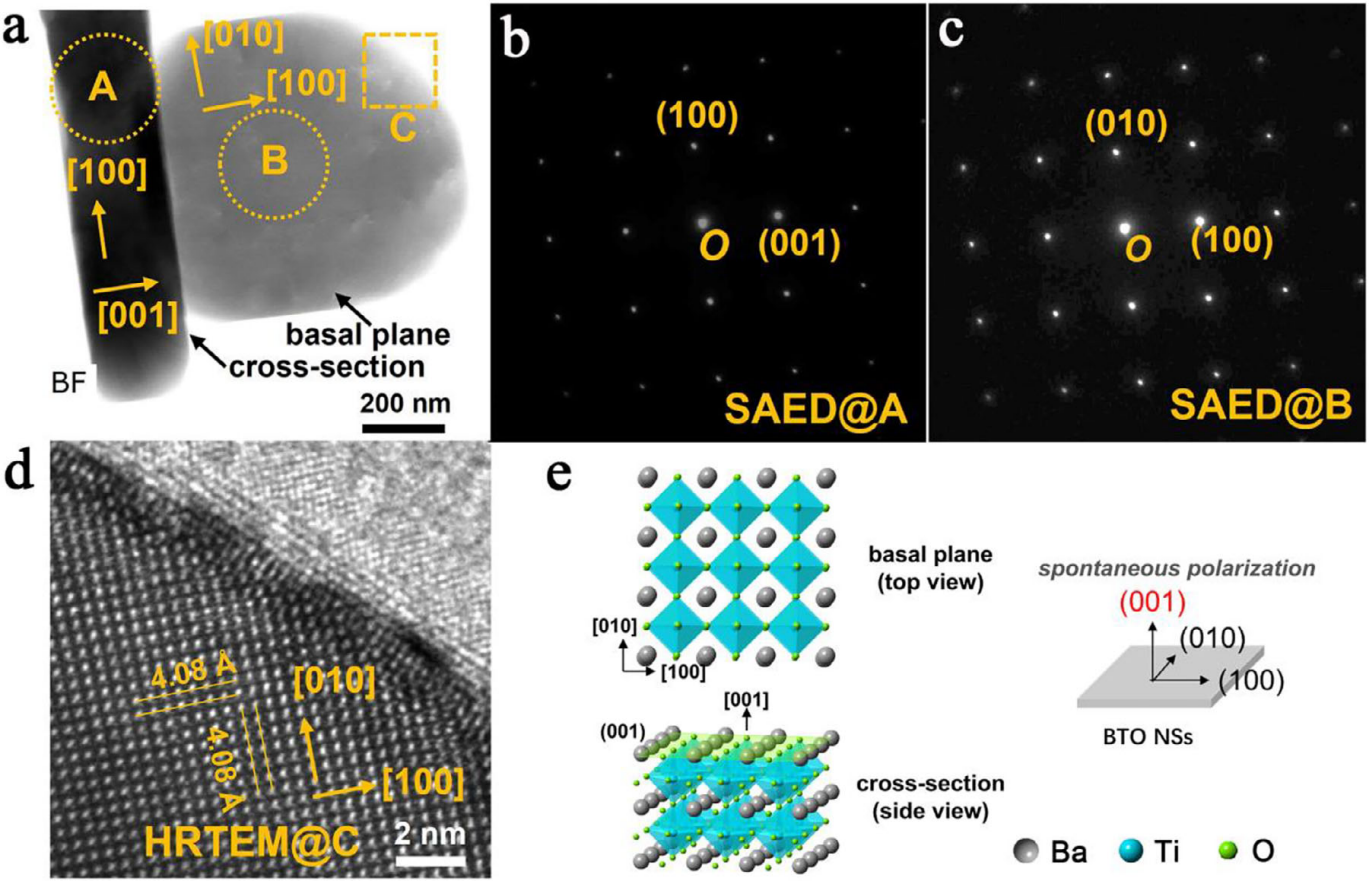
(a) TEM bright field image of BTO nanosheets. (b,c) SAED patterns corresponding to region A and region B in (a), respectively. (d) HRTEM image of region C in (a). (e) Top and side views of the crystal structure of BTO.

The high‐resolution TEM (HRTEM) technique enables direct visualization of intrinsic crystalline information through analysis of lattice fringes and selected‐area electron diffraction (SAED) patterns. To precisely determine the crystallographic orientation of the BTO nanosheets, we examined two representative specimens. The nanosheet on the left in Figure 3a shows an edge‐on (side) view, while the one on the right displays a top‐surface orientation. The enhanced contrast observed in the side‐view nanosheet originates from the increased electron scattering caused by its larger planar dimensions relative to the thickness direction. To determine the crystallographic orientations, SAED patterns (Figure 3b,c) were acquired from regions A and B (denoted by dashed circles in Figure 3a). The electron diffraction pattern revealed that in region A, the (001) crystal plane is oriented perpendicular to the thickness direction of the nanosheet, whereas in region B, the (100) and (010) planes are oriented perpendicular to the incident z‐axis. The HRTEM imaging of region C (top view) reveals well‐defined lattice fringe spacings of approximately 4.08 Å, which correspond well with the theoretical interplanar distances of the (010) and (100) planes in tetragonal BaTiO_3_. By comparing with the theoretical crystal structure of BaTiO_3_ (Figure 3e), we can conclusively determine that the main primary exposed surface of the synthesized BTO nanosheets corresponds to the (001) facet, with the polarization vector‐oriented perpendicular to the thickness direction. From a structural perspective, the pyroelectricity of BaTiO_3_ originates from the temperature‐induced off‐center displacement of Ti^4+^ cations and oxygen ion centers along the *c* axis. Therefore, BTO nanosheets with predominantly exposed (001) facets are expected to exhibit strong pyroelectric polarization along the out‐of‐plane direction. In contrast, BTO nanoparticles primarily expose (110) and (111) facets, with only a limited degree of (001) plane exposure, which may result in weaker pyroelectric polarization vectors compared to their (001)‐faceted counterparts.

Typically, the magnitude of the pyroelectric effect is fundamentally governed by the polarization vector, as quantitatively described by the pyroelectric coefficient (*p*) in the following relationship:

$$p=\frac{dP_{s}}{dT}$$

where *P*_s_ represents the spontaneous polarization and *T* denotes temperature. It is reasonable to infer that the extensive exposure of (001) facets in BTO nanosheets creates an optimal polarization orientation, thereby significantly enhances their pyroelectric response. In other words, under identical thermal excitation conditions, BTO nanosheets with highly exposed (001) facets exhibit superior pyroelectric performance compared to their nanoparticle counterparts.

**FIGURE 4:**
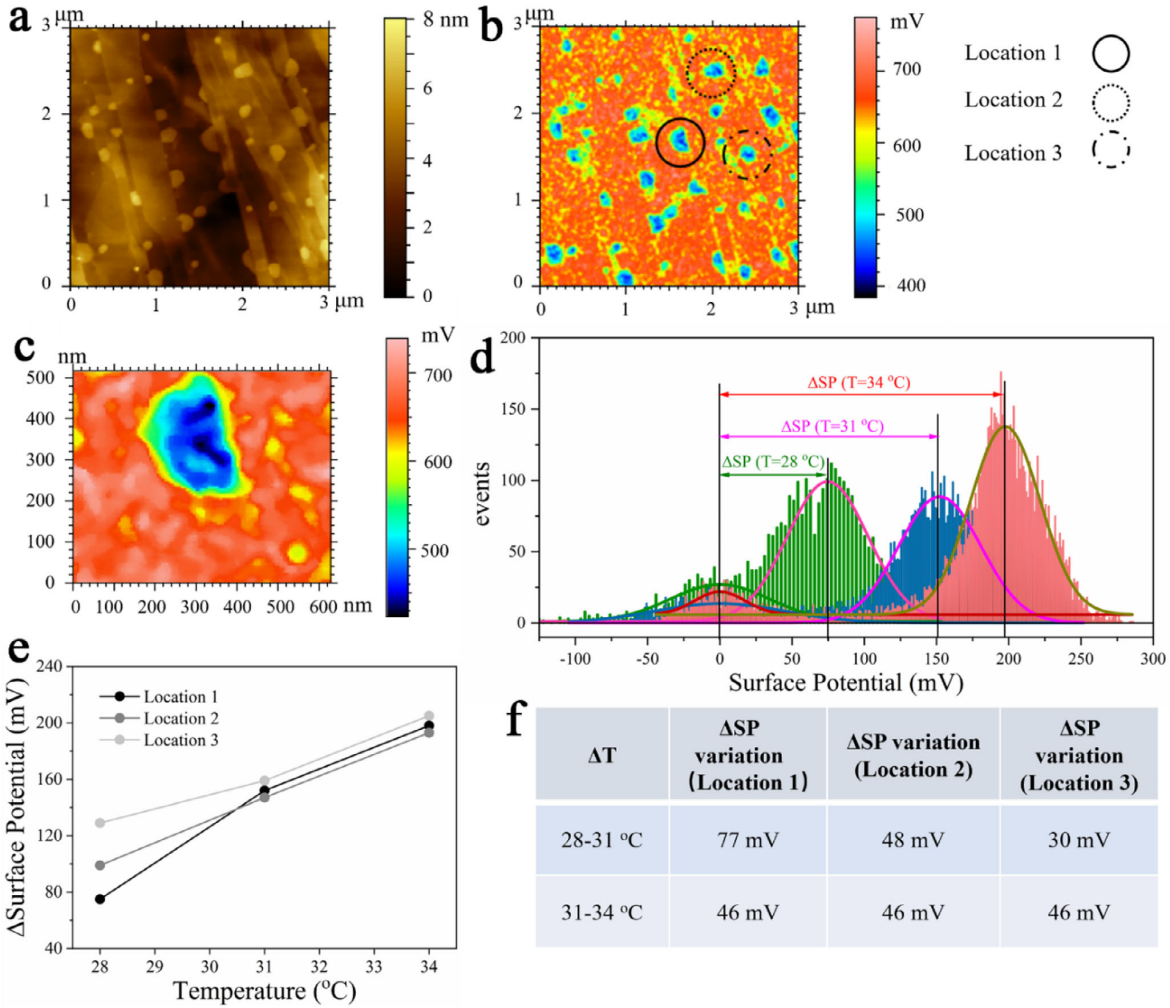
(a) Topography and (b) surface potential image of BTO nanosheets on HOPG at 31°C. (c) Magnified surface potential image of an individual BTO nanosheet at Location 3. (d) Statistical analysis of surface potential at Location 3 under different temperature conditions. (e) Temperature‐dependent surface potential variations of BTO nanosheets at different locations. (f) Surface potential differences at Locations 1, 2, and 3 during the temperature transitions from 31 °C to 28 °C and from 34 °C to 31 °C.

To visually illustrate the local pyroelectric stimulation of BTO nanosheets, their pyroelectric response was investigated using a Kelvin probe force microscope (KPFM), as depicted in Figure 4. Prior to measurements, the BTO nanosheets were dispersed in ethanol and drop‐casted onto a highly oriented pyrolytic graphite (HOPG) substrate. The topographic images in Figure 4a reveal well‐defined nanosheet morphologies with sizes in the range of several hundred nanometers, which agree well with previous SEM and TEM observations. During surface potential measurements, a temperature controller stage was employed to heat the samples incrementally, starting from 28°C, progressing to 31°C, and ultimately reaching 34°C. Figure 4b presents the corresponding surface potential distribution at 31°C, where the blue regions correspond to the BTO nanosheets and the red regions represent the HOPG substrate. For comprehensive statistical analysis, three spatially distinct nanosheets (designated as Location 1, Location 2, and Location 3) were selected. Figure 4c shows the surface potential distribution of a single BTO nanosheet in Location 1. To accurately quantify the surface potential variation (ΔSP) of BTO, we subtracted the surface potential of HOPG as a reference. It was observed that the surface potential of BTO at different temperatures follows a Gaussian distribution and increases with rising temperature (Figure 4d). This can be attributed to the fact that, as the temperature increases, the electric dipoles in BTO oscillate over a broader range relative to the polarization axis, resulting in an overall decrease in average polarization. This, in turn, leads to a decrease in surface charge compensation, thereby increasing the potential difference between BTO and HOPG. Figure 4e compares the surface potential at Locations 1, 2, and 3 under varying temperatures. It can be observed that ΔSP at all three locations increases with rising temperature, indicating a pronounced pyroelectric effect. The changes in ΔSP across two temperature ranges (28–31°C and 31–34°C) are quantitatively compared in Figure 4f, where the specific ΔSP are provided. Within the 28–31°C range, ΔSP exhibits distinct variations at different positions, as evidenced by differing slope segments. This behavior can be attributed to thermal instability. Specifically, at 28 °C, which corresponds to the initial stage of the measurement, the sample and the testing platform may not yet reached thermal equilibrium, resulting in noticeable thermal drift. After temperature adjustment and associated drift, the system may gradually reach a stable thermal equilibrium, thereby reducing deviations in the measured surface potential. Overall, KPFM effectively characterizes the pyroelectric capabilities of BTO, demonstrating a temperature‐dependent response of ΔSP/ΔT = 16.3 ± 2.1 mV °C^−1^.

**FIGURE 5:**
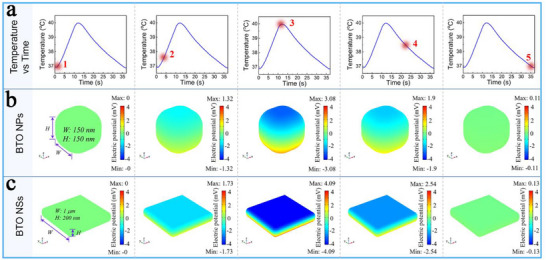
(a) Temperature variation of CNT@PDMS/BTO scaffold under illumination. (b,c) FEM simulations of the surface electric potential for BTO NPs and NSs, respectively.

Subsequently, finite element method (FEM) simulations were performed using COMSOL Multiphysics to evaluate the surface pyroelectric potential of BTO samples activated by the photothermal effect. As shown in Figure 5a, the first row displays the temporal temperature profile of the CNT@PDMS/BTO film. Upon illumination, the film rapidly heats as a result of the photothermal effect; once illumination is blocked, the film gradually cools toward ambient temperature. Accordingly, the temperature was cycled between 37°C and 40°C, with the heating phase (37°C → 40°C) occurring within approximately 12 s and the cooling phase (40°C → 37°C) taking around 26 s. Additional temperature variation cycles are presented in Figure S2. Five key time points were selected for analysis: 0 s (37°C), 5 s (37.6°C), 12 s (40°C), 23 s (38.4°C), and 38 s (37°C). At each interval, the pyroelectric potential distributions of BTO NPs (Figure 5b) and NSs (Figure 5c) were simulated. The pyroelectric potential difference across BTO exhibits a non‐monotonic, initially increasing and then decreasing, during thermal cycling. At Location #3, where the temperature variation rate reaches its maximum, the NSs exhibit a maximum potential difference of 8.18 mV, markedly higher than the 6.18 mV observed in nanoparticles. The disparity in pyroelectric response is expected to show different levels of pyroelectric stimulation, enabling tunable output intensities to meet diverse application requirements. In contrast to conventional electrical stimulation, which typically operates at relatively high intensities, such as DC voltage up to 2 V [25], AC voltage up to 1 V [26], or pulsed inputs around 300 V [27], the wireless photothermal‐pyroelectric strategy generates millivolt‐level electric potentials. This substantially lower potential not only maintains excellent neural differentiation performance, but also substantially reduces the risks of over‐stimulation, cellular damage, and degradation at the electrode‐tissue interface.

**FIGURE 6:**
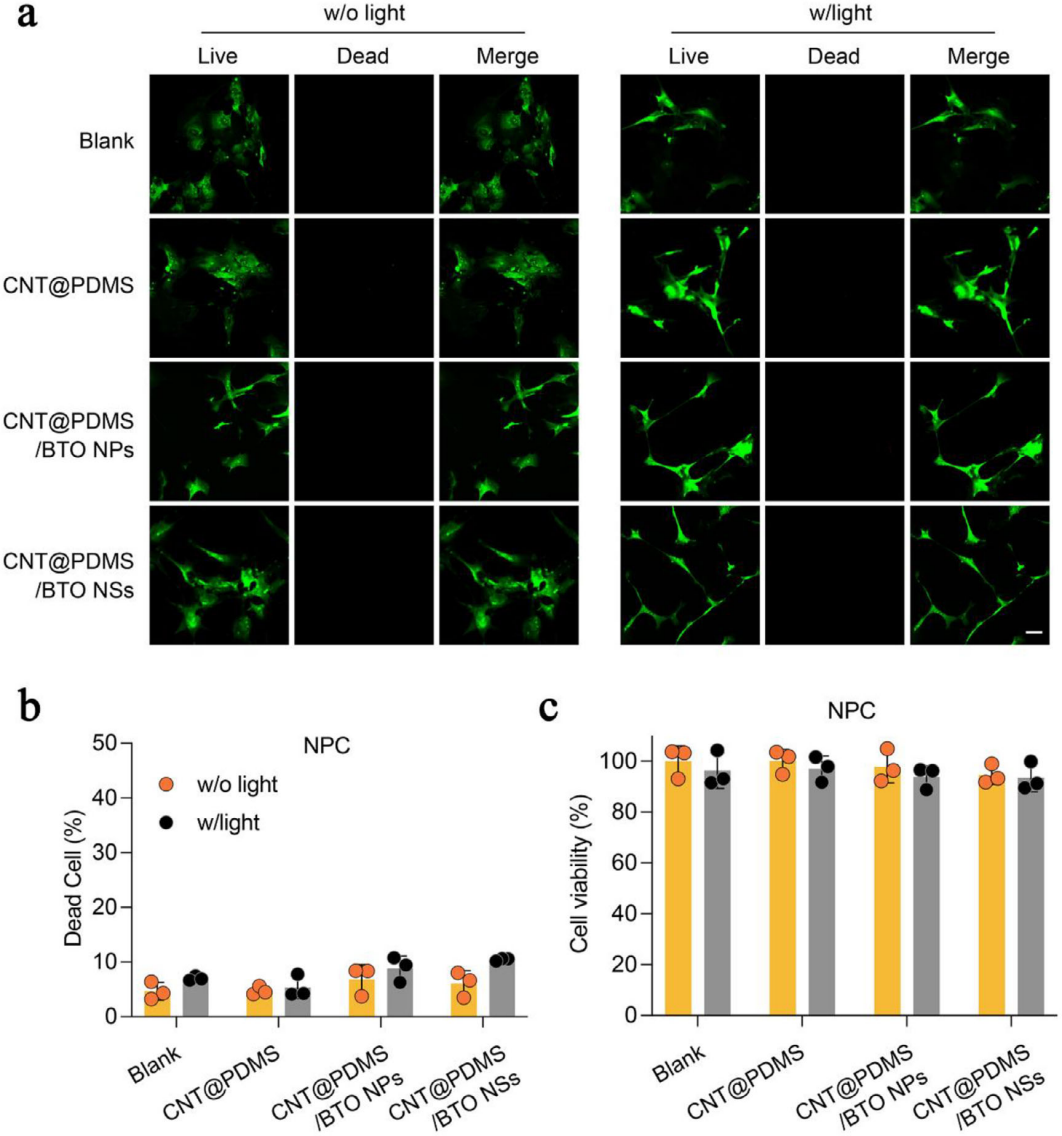
(a) Live and dead staining images of nerve cells treated with blank (as control), CNT@PDMS, CNT@PDMS/BTO NPs, and CNT@PDMS/BTO NSs under non‐illuminated (w/o light) and illuminated (w/light) conditions. Live cells are stained green, while dead cells are stained red. (b, c) Quantitative analysis of (b) the number of dead cells and (c) cell viability. n = 3 experiments, means ± s.e.m.

The differentiation potential of NPCs in response to pyroelectric stimulation was investigated by incubating NPCs with CNT@PDMS/BTO, while parallel control experiments were conducted in the absence of light exposure. Prior to differentiation studies, the biocompatibility profile of CNT@PDMS/BTO was evaluated through Live/Dead staining (Figure 6a). Regardless of light exposure or BTO presence, the live cells (green fluorescence) remained nearly unchanged after 200 photo‐thermal cycles, with minimal detection of dead cells (red fluorescence). Quantitative assessment of dead cell (Figure 6b) and neural cell viability (Figure 6c) via MTT assay revealed consistently high survival rates (>90%) across all experimental conditions, independent of illumination status. These results collectively confirm the excellent biocompatibility of CNT@PDMS/BTO composites.

**FIGURE 7:**
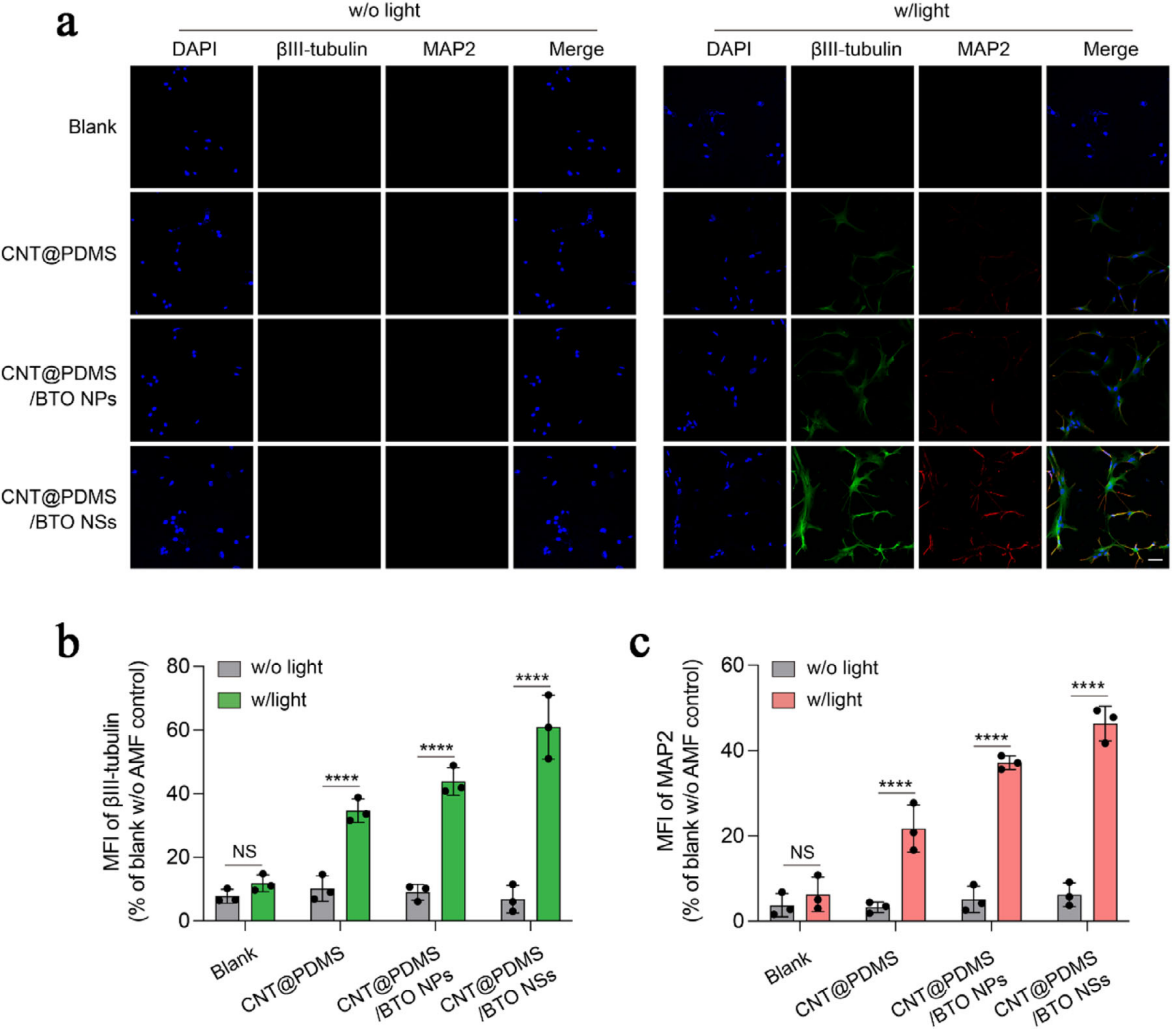
(a) Immunofluorescent staining images of DAPI, βIII‐tubulin, MAP2 and merge in progenitor cell cultured on CNT@PDMS, CNT@PDMS/BTO NPs, CNT@PDMS/BTO NSs, and control samples. (b, c) Statistical charts of protein expression for (b) βIII‐tubulin/Hoechst and (c) MAP2/Hoechst, respectively. n = 3 experiments, means ± s.e.m.

Subsequently, neural‐related protein markers were employed to evaluate the impact of pyroelectric stimulation on cell differentiation. DAPI was utilized to label cell nuclei, while βIII‐tubulin and microtubule‐associated protein 2 (MAP2) were selected as markers for early neuronal differentiation and mature neurons [28], respectively. As depicted in Figure 7a, minimal fluorescence signals for βIII‐tubulin and MAP2 were observed in the absence of light illumination or film samples. However, upon periodic light exposure, distinct fluorescence signals were observed, with their intensity following the trend: CNT@PDMS/BTO NSs > CNT@PDMS/BTO NPs > CNT@PDMS. Quantitative analysis in Figure 7b,c indicates that thermal stimulation alone induced only a low‐level expression of neural differentiation markers. In contrast, under photothermal‐pyroelectric stimulation through CNT@PDMS/BTO, the expression levels of MAP2 and βIII‐tubulin increased significantly. These immunofluorescence staining results suggest that BTO holds strong potential as an effective pyroelectric medicine for nerve regeneration. Furthermore, optimizing the out‐of‐plane polarization can improve the electrical stimulation capability of BTO, thereby enhancing cellular differentiation. It is noteworthy that, in addition to activating pyroelectric stimulation of BaTiO_3_, the mild repeated photothermal‐induced temperature fluctuations can also promote the polarization of macrophages toward the M2 phenotype, thereby enhancing anti‐inflammatory responses [29]. Moreover, previous studies have demonstrated that moderate thermal stimulation can markedly enhance endothelial cell tubulogenesis and angiogenesis, indicating a potential role in supporting tissue repair processes [30].

**FIGURE 8:**
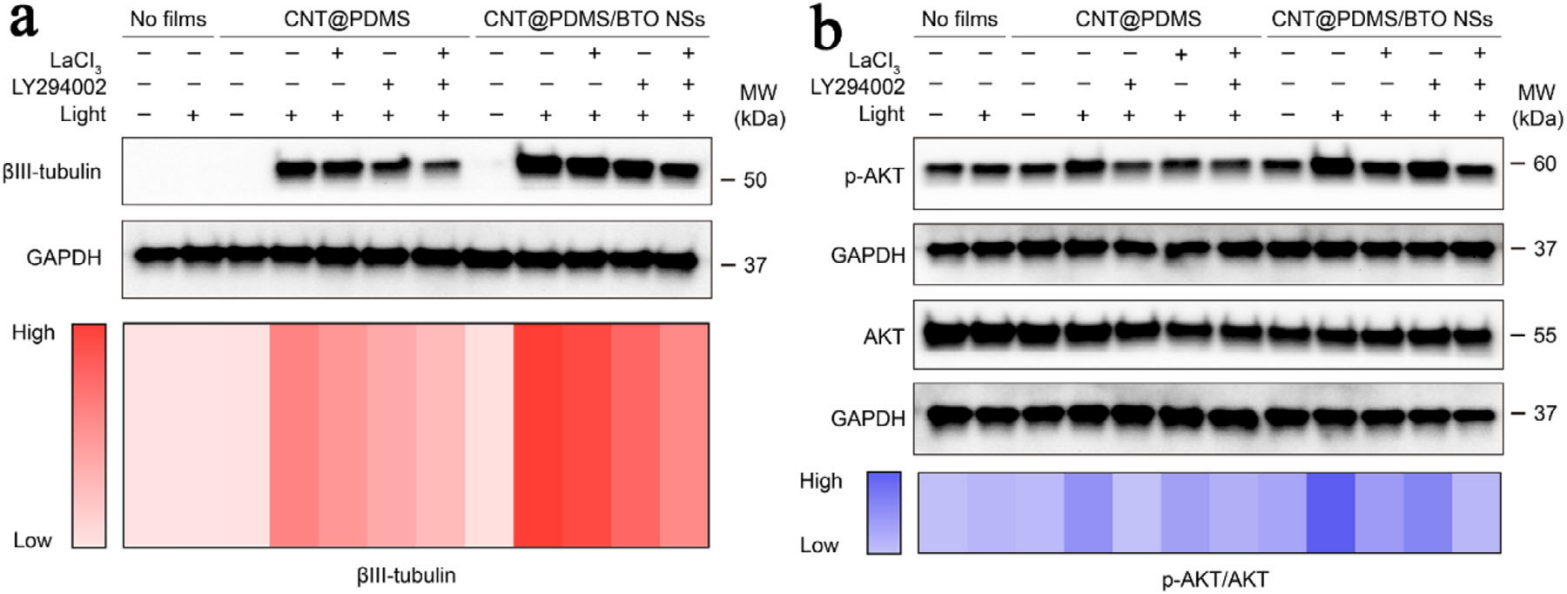
Comparative protein expression profiles of key signaling pathways across three experimental groups: control (no film), CNT@PDMS, and CNT@PDMS/BTO NSs. (a) βIII‐tubulin, and (b) p‐AKT/AKT.

To elucidate the mechanisms under differentiation promoting, we systematically investigated the effects of pyroelectric stimulation on relevant signaling pathways through systematic Western blot analysis. As shown in Figure 8, both the photothermal group (CNT@PDMS) and photothermal‐pyroelectric group (CNT@PDMS/BTO NSs) exhibited significant upregulation of βIII‐tubulin and phosphorylated AKT (P‐AKT). However, the introduction of Ca^2+^ channel blocker (LaCl_3_) and PI3K/Akt inhibitor (LY294002) markedly reduced differentiation efficiency, highlighting the critical roles of Ca^2+^ signaling and the PI3K/Akt pathway in this process. Notably, compared to the CNT@PDMS group, which provides only thermal stimulation, the CNT@PDMS/BTO NSs group, which offering both pyroelectric and thermal stimulation, exhibited a significantly higher P‐AKT/AKT ratio, indicating stronger activation of the PI3K/Akt pathway. The elevated expression of βIII‐tubulin expression further suggests enhanced cytoskeletal organization associated with neuronal differentiation. Collectively, the Western blot results demonstrate that photothermal‐pyroelectric stimulation promotes neural differentiation by simultaneously activating Ca^2+^ signaling and the PI3K/Akt pathway.

Nonetheless, since pyroelectric stimulation is inherently dependent on sustained, cyclical thermal input, it is not feasible to apply pyroelectric stimulation in the absence of a thermal effect. To isolate the influence of thermal effects on neural differentiation, we included a parallel control group exposed solely to thermal stimulation. The contribution of pure pyroelectric effect without thermal effect could be estimated by subtracting the differentiation outcome observed in the thermal‐only group from that obtained under combined thermo‐pyroelectric stimulation. It should be noted that this approach assumes a simple additive relationship between the thermal effect and pyroelectric effect and does not account for potential synergistic interactions.

To gain deeper insight into the possible synergistic mechanism between photothermal and pyroelectric stimulation, an electrical stimulation model that mimics pyroelectric stimulation without thermal excitation could be introduced. In this case, establishing experimental groups combining thermal stimulation, simulated pyroelectric stimulation, and their overlap would allow for a clearer distinction between the individual contributions of thermal and electrical stimuli, as well as the identification of any synergistic effects that may arise.

## 3 Conclusion

This study presents a novel strategy that integrates photothermal and pyroelectric effects to achieve efficient, non‐invasive electrical stimulation for neural cell differentiation. The developed CNT@PDMS/BTO platform integrates nano‐structured BaTiO_3_ (BTO) deposited onto CNT@PDMS composite films, where periodic light illumination induces localized temperature gradients that, in turn, activate the pyroelectric effect of BTO. When co‐cultured with neural stem cells, the photothermal‐pyroelectric stimulation significantly accelerates their differentiation into mature neurons. Notably, our results reveal that CNT@PDMS/BTO films incorporating BTO nanosheets with out‐of‐plane polarization exhibit significantly enhanced neurogenic differentiation compared to films containing BTO nanoparticles. These findings suggest that CNT@PDMS/BTO films hold great promise as a non‐contact pyroelectric regenerative medicine platform for non‐invasive repair of injured neural tissue.

## 4 Experimental Section

### 4.1 Preparation of BTO Nanosheets

BTO nanosheets were synthesized through a template‐assisted hydrothermal method based on our previous work [31], in which Bi_4_Ti_3_O_12_ nanosheets served as templates and a source of titanium.

Bi_4_Ti_3_O_12_ nanosheets were synthesized via a one‐step molten salt method. Specifically, 3.625 g of NaCl, 4.659 g of KCl, 2.31 g of Bi_2_O_3_, and 0.594 g of TiO_2_ (a molar ratio of NaCl : KCl : Bi_2_O_3_ : TiO_2_ = 62.5 : 62.5 : 5 : 7.5) was thoroughly mixed and ground in an agate mortar for 60 min. This homogeneous mixture was then transferred to a corundum crucible and calcined at 700°C for 2 h with a heating rate of 10°C min^−1^. After naturally cooling to room temperature, the resulting products were thoroughly washed repeatedly with deionized water and ethanol to remove residual salts, and subsequently dried at 80°C overnight.

To synthesize BTO nanosheets, a hydrothermal conversion of Bi_4_Ti_3_O_12_ was employed, wherein Bi_4_Ti_3_O_12_ served as the titanium source, and BaCl_2_·2H_2_O provided the barium source. Sodium oleate (NaOL) was introduced to regulate the morphology of BTO. First, 0.12 g of NaOL was dissolved in 160 mL of 12 M NaOH aqueous solution and stirred for 30 min. Subsequently, 2.198 g of BaCl_2_·2H_2_O and 0.4 g of Bi_4_Ti_3_O_12_ were added to the solution and stirred for another 90 min. The resulting suspension was then transferred to a 200 mL Teflon‐lined stainless‐steel autoclave and subjected to hydrothermal treatment at 240°C for 20 h. The final precipitate was collected by centrifugation, thoroughly washed with 2 M HNO_3_ solution, deionized water and ethanol, followed by drying at 80°C overnight to obtain BTO nanosheets.

### 4.2 Preparation of BTO Nanoparticles

The BTO nanoparticles (99.9%, 200 nm) were commercially obtained from US Research Nanomaterials, Inc., and used without further purification.

### 4.3 Fabrication of Photothermal‐Pyroelectric Films

To fabricate the photothermal‐pyroelectric composite films, BTO nanosheets or nanoparticles were dispersed in ethanol via ultrasonic treatment for 3 min to ensure uniform dispersion. The resulting suspension was then drop‐cast onto a silicon wafer and evenly spread using a custom‐made blader. Separately, carbon nanotubes were weighed and added to the PDMS solution, followed by continuous stirring for 30 min to obtain a homogeneous mixture. This mixture was subsequently drop‐cast onto the silicon wafer pre‐coated with BTO nanosheets or nanoparticles. The assembled wafer was then subjected to spin‐coating for 5 min to achieve a uniform film. After spin‐coating, the wafer was transferred to an oven and cured at 120°C for 20 min. Upon curing, the CNT@PDMS film embedded with BTO nanosheet or nanoparticle could be easily peeled off from the silicon substrate.

### 4.4 Materials Characterization

The crystalline phase of the samples was investigated through XRD analysis using a Bruker AXS D8 Advance diffractometer with Cu Ka radiation. SEM images were acquired using a JSM‐7100F field‐emission electron microscope. TEM and EDX analysis were performed on an FEI Talos F200X (Chem S/TEM) operated at an accelerating voltage of 200 kV. KPFM measurements were conducted using a Park NX10 scanning probe system, equipped with Si cantilevers coated with a Pt layer. The cantilever operated at a resonated frequency of 3 kHz, and an AC voltage of 1 V was applied between the probe and the sample. Before the test, BTO samples were dropped‐casted from an ethanol suspension onto the HOPG substrate.

### 4.5 Photothermal – Pyroelectric Stimulation

The composite film was cut into dimensions of 20 mm × 30 mm, appropriately labeled according to its surface composition. Then, the film samples were placed in a constant temperature and humidity chamber to maintain stable environmental conditions. To achieve consistent thermal stimulation and establish a generalizable foundation for photothermal‐pyroelectric research, a xenon lamp (MAX‐303, ASAHI‐SPECTRA, Japan) was employed. This light source provides a powerful broad‐spectrum output (385–740 nm) at an intensity of 10 mW cm^−2^ and was used to irradiate the film from the side opposite to the BTO. The illumination process consisted of cyclic 12‐s light exposure intervals alternated with 26‐s dark periods. During irradiation, the photothermal effect induced uniform heating of the film, while the absence of irradiation allowed the film to gradually cool under ambient temperature, thereby establishing a cyclic thermal gradient. Thus, the pyroelectric effect of BTO was activated, generating endogenous electric fields that facilitated the differentiation of adjacent cells.

### 4.6 FEM Simulation

FEM simulation for BTO was conducted using COMSOL Multiphysics software. The dimension of BTO nanoparticles was set to 150 nm in height and width, while those of BTO nanosheets were set to 200 nm in height and 1000 nm in width, consistent with the experimentally characterized samples. Equation 𝐷 = 𝜀_0_𝜀_r_ + 𝑝(𝑇 − 𝑇_0_) was used in the Electric Current (ec) interface under the AC/DC branch, where 𝑝 represents the pyroelectric coefficient. The polar axes of the modeled BTO were aligned along the z‐axis by default. The 𝑝 values along the polar axis of BTO was set to −20 µC·m^−2^·K^−1^ according to the literature [32]. Additional material parameters, including the elasticity matrix, coupling matrix, and relative permittivity of BTO, were obtained from the predefined material parameters in Comsol Multiphysics.

### 4.7 Human iPSC Derived Neural Progenitor Cell (NPC) Culture

In the cultivation of human iPSC‐derived neural progenitor cells, Matrigel‐coated culture wares were essential. Matrigel was thawed at low temperatures and diluted 1:50 with cold DMEM medium before being applied to 6‐well plates and culture flasks. The coated vessels were incubated at room temperature for 1 h or overnight at 2–8°C to allow proper adhesion. Before cell seeding, the coating solution was removed, and ENStem‐A Neural Expansion Medium was added. Cryopreserved cells were rapidly thawed in a 37°C water bath, transferred to a sterile conical tube, and gently mixed with pre‐warmed medium to prevent osmotic shock. Following centrifugation and supernatant removal, cells were resuspended, counted for viability, and plated onto the prepared Matrigel‐coated culture ware. The culture was maintained at 37°C in a 5% CO_2_ humidified incubator, with medium changes every 2–3 days. Upon reaching confluence, cells were dissociated with Accutase, counted, and replated for further expansion in T75 flasks prepared similarly, ensuring optimal conditions for neural progenitor proliferation and maintenance.

### 4.8 Cell Viability Tests

Cell viability assessments were performed utilizing NPCs. Composite films with or without BTO samples were sterilized under ultraviolet (UV) radiation for 3 h. Subsequently, these films were submerged in a 10% solution of penicillin‐streptomycin and Amphotericin B (sourced from Sigma Aldrich) for antimicrobial treatment, then extensively rinsed with phosphate‐buffered saline (PBS). The prepared samples were then allocated to 6‐well plates to facilitate cell culture. NPCs were inoculated at a seeding density of 3 × 10^6^ cells per well for 72‐h viability analysis using 3‐(4,5‐dimethylthiazol‐2‐yl)‐2,5‐diphenyltetrazolium bromide (MTT) assays. Then, the CNT@PDMS, CNT@PDMS/BTO NPs, and CNT@PDMS/BTO NSs were tested under the above‐mentioned photo‐thermal process for 200 cycles. The stimulation was conducted over two days, with 100 cycles administered per day. Each cycle consisted of a 12‐s light exposure interval followed by a 26‐s dark period. After the first 100 stimulation cycles, the cells and film samples were returned to the cell culture incubator for a 24‐h incubation period. This was followed by the administration of the second set of 100 photothermal‐pyroelectric stimulation cycles. After 72 h of incubation, MTT reagent (3 mg mL^−1^), was applied to each well. Quantification of sample absorbance at a wavelength of 570 nm (with a reference wavelength of 630 nm) was conducted using the Varioskan Flash multimode microreader, and the mean value derived from triplicate wells was determined.

Following standardized protocols, the biocompatibility was also explored after 72 h incubation by the live/dead assay. In the evaluation of cell viability, cells underwent incubation with a live/dead assay solution (Invitrogen, Catalogue Number: R37601) for a duration of 15 min at ambient temperature. Subsequently, the specimens were visualized using a confocal microscope (LSM 880 Airyscan, Zeiss), and the images obtained were analyzed quantitatively with the aid of ImageJ software.

### 4.9 Immunofluorescent Staining

To assess the pyroelectric stimulatory effects of BTO under consistent photothermal‐pyroelectric cycling, NPCs were seeded in ibidi 3 Well Chamber removable slides product number 80381 at 1.0 × 10^5^ cells per well and cultured under defined light/dark cycles for 4 days and then fixed in 4% paraformaldehyde (PFA) for 10 min at ambient temperature. Following fixation, cells were permeabilized using 0.5% Triton X‐100 for 30 min, and non‐specific binding was blocked by 5% goat serum for 30 min. Primary antibody staining was performed overnight at 4°C using βIII‐tubulin (Sigma–Aldrich, T2200) and microtubule‐associated protein 2 (MAP2) (Proteintech, 67015‐1) to identify neuronal differentiation markers. After thorough washing, samples were incubated for 1 h at room temperature with secondary antibodies: FITC‐conjugated goat anti‐rabbit IgG (Sigma–Aldrich, AP132F) and TRITC‐conjugated goat anti‐mouse IgG (Sigma–Aldrich, AP503R). This was followed by a series of washes and nuclear staining. For each condition, confocal images were acquired from randomly selected fields of view across the entire well in three independent differentiation experiments (n = 3), and all quantified data were subjected to statistical analysis as described in the statistical analysis section. Visualization of the stained samples was achieved through confocal laser scanning microscopy (Zeiss LSM 880 Airyscan), with subsequent image quantification conducted via ImageJ software.

### 4.10 Western Blot Analysis

For the Western blot assay, proteins were extracted from cultured cells using Thermo Scientific RIPA Lysis and Extraction Buffer (Catalog number: 89900), supplemented with PMSF Protease Inhibitor (Catalog number: 36978) to enhance protein yield and prevent proteolytic degradation. Protein concentrations were quantified using the Thermo Scientific Pierce BCA Protein Assay Kit (Catalog number: 23227). Separation of proteins was performed through SDS‐PAGE using Tris‐Glycine‐SDS Buffer (Sigma, T7777) at the recommended working concentration. Following electrophoresis, proteins were transferred onto polyvinylidene fluoride (PVDF) membranes using Thermo Scientific Pierce 10X Western Blot Transfer Buffer, Methanol‐free (Catalog number: 35040). To reduce nonspecific binding, membranes were blocked with Thermo Scientific Pierce Clear Milk Blocking Buffer (10X), diluted to 1X (Catalog number: 37587), for one hour at room temperature. Membranes were subsequently incubated overnight at 4°C with primary antibodies targeting βIII‐tubulin (Sigma, T2200), AKT (Proteintech, 10176‐2‐AP), and Phospho‐AKT (Proteintech, 80455‐1‐RR) using the manufacturers’ recommended dilutions. After primary antibody incubation, membranes were washed and incubated with horseradish peroxidase (HRP)‐conjugated secondary antibodies (Sigma, AP307P) for one hour at room temperature. Protein bands were detected using Clarity Western ECL Substrate (Bio‐Rad, 200 mL #1705060) and visualized using a Bio‐Rad imaging system (ChemiDoc XRS+). Digital images were analyzed and quantified using ImageJ software.

### 4.11 Statistical Analysis

Immunofluorescence data are presented as mean ± standard error of the mean (s.e.m.). Differences among multiple groups were evaluated using one way analysis of variance (ANOVA) followed by Tukey post hoc tests. All statistical analyses were performed using GraphPad Prism version 9.5.0 (GraphPad Software, 2022). Values of p < 0.05 were considered statistically significant.

## Conflicts of Interest

The authors declare no conflicts of interest.

## Supporting information




**Supporting File**: advs73628‐sup‐0001‐SuppMat.docx.

## Data Availability

The data that support the findings of this study are available in the supplementary material of this article.

## References

[1] T. Mollayeva , S. Mollayeva , and A. Colantonio , “Traumatic Brain Injury: Sex, Gender and Intersecting Vulnerabilities,” Nature Reviews Neurology 14 (2018): 711–722, 10.1038/s41582-018-0091-y. 30397256

[2] M. T. Heemels , “Neurodegenerative Diseases,” Nature 539 (2016): 179, 10.1038/539179a. 27830810

[3] M. Vassal , F. Martins , B. Monteiro , S. Tambaro , R. Martinez‐Murillo , and S. Rebelo , “Emerging Pro‐neurogenic Therapeutic Strategies for Neurodegenerativediseases: A Review of Pre‐clinical and Clinical Research,” Molecular Neurobiology 62 (2025): 46–76, 10.1007/s12035-024-04246-w. 38816676 PMC11711580

[4] Z. A. Sun , H. Q. Hu , X. C. Zhang , et al., “Recent Advances in Peptide‐based Bioactive Hydrogels for Nerve Repair and Regeneration: From Material Design to Fabrication, Functional Tailoring and Applications,” Journal of Materials Chemistry B 12 (2024): 2253–2273, 10.1039/D4TB00019F. 38375592

[5] C. E. Schmidt and J. B. Leach , “Neural Tissue Engineering: Strategies for Repair and Regeneration,” Annual Review of Biomedical Engineering 5 (2003): 293–347, 10.1146/annurev.bioeng.5.011303.120731. 14527315

[6] L. L. Liang , C. H. Sun , R. T. Zhang , et al., “Piezotronic Effect Determined Neuron‐Like Differentiation of Adult Stem Cells Driven by Ultrasound,” Nano Energy 90 (2021): 106634, 10.1016/j.nanoen.2021.106634.

[7] I. Fischer , J. N. Dulin , and M. A. Lane , “Transplanting Neural Progenitor Cells to Restore Connectivity After Spinal Cord Injury,” Nature Reviews Neuroscience 21 (2020): 366–383, 10.1038/s41583-020-0314-2. 32518349 PMC8384139

[8] T. Kitagawa , N. Nagoshi , H. Okano , and M. Nakamura , “A Narrative Review of Advances in Neural Precursor Cell Transplantation Therapies for Spinal Cord Injury,” Neurospine 19 (2022): 935–945, 10.14245/ns.2244628.314. 36597632 PMC9816589

[9] B. H. Shan and F. G. Wu , “Hydrogel‐Based Growth Factor Delivery Platforms: Strategies and Recent Advances,” Advanced Materials 36 (2024): 2210707, 10.1002/adma.202210707. 37009859

[10] M. R. Love , S. Palee , S. C. Chattipakorn , and N. Chattipakorn , “Effects of Electrical Stimulation on Cell Proliferation and Apoptosis,” Journal of Cellular Physiology 233 (2018): 1860–1876, 10.1002/jcp.25975. 28452188

[11] J. Guo , J. Cao , J. H. Wu , and J. Q. Gao , “Electrical Stimulation and Conductive Materials: Electrophysiology‐based Treatment for Spinal Cord Injury,” Biomaterials Science 12 (2024): 5704–5721, 10.1039/D4BM00959B. 39403758

[12] H. Cheng , Y. Huang , H. Q. Yue , Y. B. Fan , and A. Faroni , “Electrical Stimulation Promotes Stem Cell Neural Differentiation in Tissue Engineering,” Stem Cells International 2021 (2021): 6697574. 33968150 10.1155/2021/6697574 PMC8081629

[13] S. B. Rajendran , K. Challen , K. L. Wright , and J. G. Hardy , “Electrical Stimulation to Enhance Wound Healing,” Journal of Functional Biomaterials 12 (2021): 40, 10.3390/jfb12020040. 34205317 PMC8293212

[14] S. Preetam , A. Ghosh , R. Mishra , et al., “Electrical Stimulation: A Novel Therapeutic Strategy to Heal Biological Wounds,” RSC Advances 14 (2024): 32142–32173. 39399261 10.1039/d4ra04258a PMC11467653

[15] N. Rossetti , J. Hagler , P. Kateb , and F. Cicoira , “Neural and Electromyography PEDOT Electrodes for Invasive Stimulation and Recording,” Journal of Materials Chemistry C 9 (2021): 7243–7263, 10.1039/D1TC00625H.

[16] X. Yuan , J. C. Shi , Y. Kang , J. R. Dong , Z. C. Pei , and X. Y. Ji , “Piezoelectricity, Pyroelectricity, and Ferroelectricity in Biomaterials and Biomedical Applications,” Advanced Materials 36 (2024): 2308726. 10.1002/adma.202308726 37842855

[17] W. J. Wang , J. H. Li , H. Liu , and S. H. Ge , “Advancing Versatile Ferroelectric Materials Toward Biomedical Applications,” Advanced Science 8 (2021): 2003074, 10.1002/advs.202003074. PMC7788502 33437585

[18] A. Sood , M. Desseigne , A. Dev , et al., “A Comprehensive Review on Barium Titanate Nanoparticles as a Persuasive Piezoelectric Material for Biomedical Applications: Prospects and Challenges,” Small 19 (2023): 2206401, 10.1002/smll.202206401. 36585372

[19] K. K. Das , B. Basu , P. Maiti , and A. K. Dubey , “Interplay of Piezoelectricity and Electrical Stimulation in Tissue Engineering and Regenerative Medicine,” Applied Materials Today 39 (2024): 102332, 10.1016/j.apmt.2024.102332.

[20] A. Cafarelli , A. Marino , L. Vannozzi , et al., “Piezoelectric Nanomaterials Activated by Ultrasound: The Pathway From Discovery to Future Clinical Adoption,” ACS Nano 15 (2021): 11066–11086, 10.1021/acsnano.1c03087. 34251189 PMC8397402

[21] M. Hoogenboom , D. Eikelenboom , M. H. Brok , A. Heerschap , J. J. Fütterer , and G. J. Adema , “Mechanical High‐intensity Focused Ultrasound Destruction of Soft Tissue: Working Mechanisms and Physiologic Effects,” Ultrasound in Medicine & Biology 41 (2015): 1500–1517, 10.1016/j.ultrasmedbio.2015.02.006. 25813532

[22] H. C. Liu , G. C. Huang , R. Wang , et al., “Carbon Nanotubes Grown on the Carbon Fibers to Enhance the Photothermal Conversion Toward Solar‐driven Applications,” ACS Applied Materials & Interfaces 14 (2022): 32404–32411, 10.1021/acsami.2c07970. 35796654

[23] M. D. Min , Y. M. Liu , C. Y. Song , et al., “Photothermally Enabled Pyro‐catalysis of a BaTiO_3_ Nanoparticle Composite Membrane at the Liquid/Air Interface,” ACS Applied Materials & Interfaces 10 (2018): 21246–21253, 10.1021/acsami.8b03411. 29870218

[24] J. Wu , H. Ye , E. Zhang , et al., “BaTiO_3_ Nanocrystals With Tunable Exposed {001} Polar Facets: a High‐performance Piezocatalyst and Piezoelectric Regenerative Medicine,” Nano Energy 130 (2024): 110115, 10.1016/j.nanoen.2024.110115.

[25] G. Thrivikraman , G. Madras , and B. Basu , “Intermittent Electrical Stimuli for Guidance of human Mesenchymal Stem Cell Lineage Commitment towards Neural‐Like Cells on Electroconductive Substrates,” Biomaterials 35 (2014): 6219–6235, 10.1016/j.biomaterials.2014.04.018. 24816362

[26] M. A. Matos and M. T. Cicerone , “Alternating Current Electric Field Effects on Neural Stem Cell Viability and Differentiation,” Biotechnology Progress 26 (2010): 664–670, 10.1002/btpr.389. 20205161

[27] W. B. Guo , X. D. Zhang , X. Yu , et al., “Self‐Powered Electrical Stimulation for Enhancing Neural Differentiation of Mesenchymal Stem Cells on Graphene–Poly(3,4‐ethylenedioxythiophene) Hybrid Microfibers,” ACS Nano 10 (2016): 5086–5095, 10.1021/acsnano.6b00200. 27144593

[28] L. Wang , H. Zhao , M. Han , et al., “Electromagnetic Cellularized Patch With Wirelessly Electrical Stimulation for Promoting Neuronal Differentiation and Spinal Cord Injury Repair,” Advanced Science 11 (2024): 2307527, 10.1002/advs.202307527. 38868910 PMC11321663

[29] F. J. Dai , J. Y. Zhang , F. J. Chen , et al., “A Multi‐Responsive Hydrogel Combined with Mild Heat Stimulation Promotes Diabetic Wound Healing by Regulating Inflammatory and Enhancing Angiogenesis,” Advanced Science 11 (2024): 2408783, 10.1002/advs.202408783. 39435670 PMC11633493

[30] Y. Yuan , D. D. Fan , S. H. Shen , and X. X. Ma , “An M2 Macrophage‐polarized Anti‐inflammatory Hydrogel Combined With Mild Heat Stimulation for Regulating Chronic Inflammation and Impaired Angiogenesis of Diabetic Wounds,” Chemical Engineering Journal 433 (2022): 133859, 10.1016/j.cej.2021.133859.

[31] Q. Tang , J. Wu , D. Kim , et al., “Enhanced Piezocatalytic Performance of BaTiO_3_ Nanosheets with Highly Exposed {001} Facets,” Advanced Functional Materials 32 (2022): 2202180, 10.1002/adfm.202202180.

[32] W. Q. Qian , H. T. Wu , and Y. Yang , “Ferroelectric BaTiO_3_ Based Multi‐Effects Coupled Materials and Devices,” Advanced Electronic Materials 8 (2022): 2200190, 10.1002/aelm.202200190.

