# Supplementary material for: A Soft, Flexible Implant for Wireless Photothermal–Pyroelectric Neurostimulation
Source: Adv Sci (Weinh). 2026 Jan 20:e19616. Online ahead of print. doi: 10.1002/advs.202519616 (PMC13325507; doi:10.1002/advs.202519616)
Supplement: Supplementary file 1 — Supporting File: advs73628‐sup‐0001‐SuppMat.docx. [file ADVS-9999-e19616-s001.docx]

Supporting Information

**A Soft, Flexible Implant for Wireless Photothermal–Pyroelectric Neurostimulation**

Jiang Wu, Minmin Mao*, Beltzane Garcia Cirera, Hao Ye*, Xiangzhong Chen, Josep Puigmartí-Luis*, Ni Qin, Salvador Pané


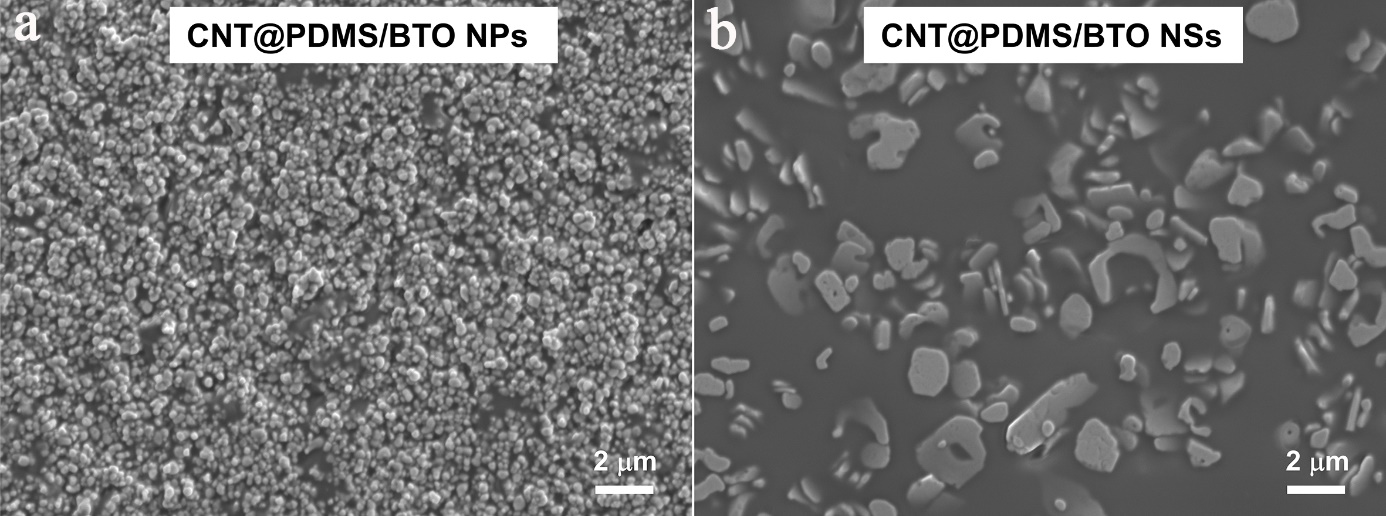


**Figure S1** The SEM images of CNT@PDMS scaffold loaded with BTO on the surface in lower magnification: a) CNT@PDMS/ BTO NPs and (b) CNT@PDMS/ BTO NSs.





**Figure S2** Real-time temperature profile over ten cycles during cell experiments.
